# Reversing the directionality of reactions between non-oxidative pentose phosphate pathway and glycolytic pathway boosts mycosporine-like amino acid production in *Saccharomyces cerevisiae*

**DOI:** 10.1186/s12934-024-02365-6

**Published:** 2024-05-09

**Authors:** Miselle Tiana Hengardi, Cui Liang, Keshiniy Madivannan, Lay Kien Yang, Lokanand Koduru, Yoganathan Kanagasundaram, Prakash Arumugam

**Affiliations:** 1 Agency for Science, Technology and Research (A*STAR), Singapore Institute of Food and Biotechnology Innovation, 31 Biopolis Way, Singapore, 138869 Singapore; 2 NUS Graduate School for Integrated Sciences and Engineering, National University of Singapore, 21 Lower Kent Ridge Road, Singapore, 119077 Singapore; 3 Antimicrobial Resistance Interdisciplinary Research Group, Singapore-MIT Alliance for Research and Technology, 1 CREATE Way, Singapore, 138602 Singapore; 4 Innovation & Enterprise, Agency for Science, Technology and Research (A*STAR), 1 Fusionopolis Way, Singapore, 138632 Singapore; 5 Institute of Molecular and Cell Biology, Agency for Science, Technology and Research (A*STAR), 61 Biopolis Drive, Singapore, 138673 Singapore; 6 School of Biological Sciences, Nanyang Technological University, Singapore, 637551 Singapore

**Keywords:** *Saccharomyces cerevisiae*, Metabolic engineering, Mycosporine-like amino acids, Metabolic pathway, Reversed non-oxidative pentose phosphate pathway

## Abstract

**Background:**

Mycosporine-like amino acids (MAAs) are a class of strongly UV-absorbing compounds produced by cyanobacteria, algae and corals and are promising candidates for natural sunscreen components. Low MAA yields from natural sources, coupled with difficulties in culturing its native producers, have catalyzed synthetic biology-guided approaches to produce MAAs in tractable microbial hosts like *Escherichia coli*, *Saccharomyces cerevisiae* and *Corynebacterium glutamicum*. However, the MAA titres obtained in these hosts are still low, necessitating a thorough understanding of cellular factors regulating MAA production.

**Results:**

To delineate factors that regulate MAA production, we constructed a shinorine (mycosporine-glycine-serine) producing yeast strain by expressing the four MAA biosynthetic enzymes from *Nostoc punctiforme* in *Saccharomyces cerevisiae*. We show that shinorine is produced from the pentose phosphate pathway intermediate sedoheptulose 7-phosphate (S7P), and not from the shikimate pathway intermediate 3-dehydroquinate (3DHQ) as previously suggested. Deletions of transaldolase (*TAL1*) and phosphofructokinase (*PFK1*/*PFK2*) genes boosted S7P/shinorine production via independent mechanisms. Unexpectedly, the enhanced S7P/shinorine production in the *PFK* mutants was not entirely due to increased flux towards the pentose phosphate pathway. We provide multiple lines of evidence in support of a reversed pathway between glycolysis and the non-oxidative pentose phosphate pathway (NOPPP) that boosts S7P/shinorine production in the phosphofructokinase mutant cells.

**Conclusion:**

Reversing the direction of flux between glycolysis and the NOPPP offers a novel metabolic engineering strategy in *Saccharomyces cerevisiae*.

**Supplementary Information:**

The online version contains supplementary material available at 10.1186/s12934-024-02365-6.

## Background

Mycosporines and mycosporine-like amino acids (MAAs) are small molecules which strongly absorb ultraviolet radiation (UVR) in the range from 310 to 360 nm and are typically produced by cyanobacteria, fungi, and algae [1]. These compounds have stimulated immense commercial interest due to their potential as natural, safe and eco-friendly sunscreens. Existing suncare products contain a UV blocker in the form of either a physical or a chemical sunscreen. Physical sunscreens such as zinc oxide and titanium dioxide reflect UV light, while chemical sunscreens such as avobenzone and octosilate absorb UV light and dissipate the energy in the form of heat. However, these sunscreens are unsuitable for long-term use as they cause harmful effects such as endocrine disruption, genotoxicity, coral bleaching, as well as other undesirable consequences on human health and the environment [2]. Hence, there is great interest in finding alternative bio-based sunscreens with no accompanying negative effects. Among these, MAAs are particularly interesting as they not only strongly absorb UV radiation, but also display anti-inflammatory, anti-oxidant, and anti-ageing activities [3–5].

Mycosporines comprise a cyclohexenone ring with a ketone at C1 and an amino compound at C3, while MAAs possess an imino-substituent at C1 instead [6]. There are more than 30 MAAs that have been reported to occur naturally [7]. While primary MAAs differ in the type of amino acid substituents attached to the cyclohexenone ring, and absorb UV maximally in the 330–335 nm range, secondary MAAs are produced through modifications to the side chains of primary MAAs. These processes could include esterification, amidation, dehydration, decarboxylation, and nitrogen substitution [8, 9]. A few secondary MAAs such as palythene and usujirene absorb maximally at about 360 nm [10].

Shinorine, an MAA with glycine and serine substituents, has a high molar extinction coefficient (ε = 44,668 M^− 1^cm^− 1^) and absorption maximum at 334 nm [11], making it an attractive sunscreen molecule. However, the low titers of shinorine in its natural producers limits its utility as a commercialization target, suggesting that its production in a heterologous host could be a promising alternative [12]. *Escherichia coli* [13], *Corynebacterium glutamicum* [14] and *Saccharomyces cerevisiae* [15, 16] have been successfully used as heterologous hosts for shinorine production, but the yields remain low (< 200 mg/L) [17]. Methods to increase MAA yields in heterologous hosts include the use of xylose as a co-substrate [16], the deletion of the hexose kinase *HXK2* to reduce glycolytic flux, as well as bioprocess engineering [18]. The highest reported titer in a heterologous producer at this time is 1.53 g/L of shinorine from an engineered strain of *S. cerevisiae* [19], indicating the potential of baker’s yeast as a host for large scale production of MAAs.

**Fig. 1:**
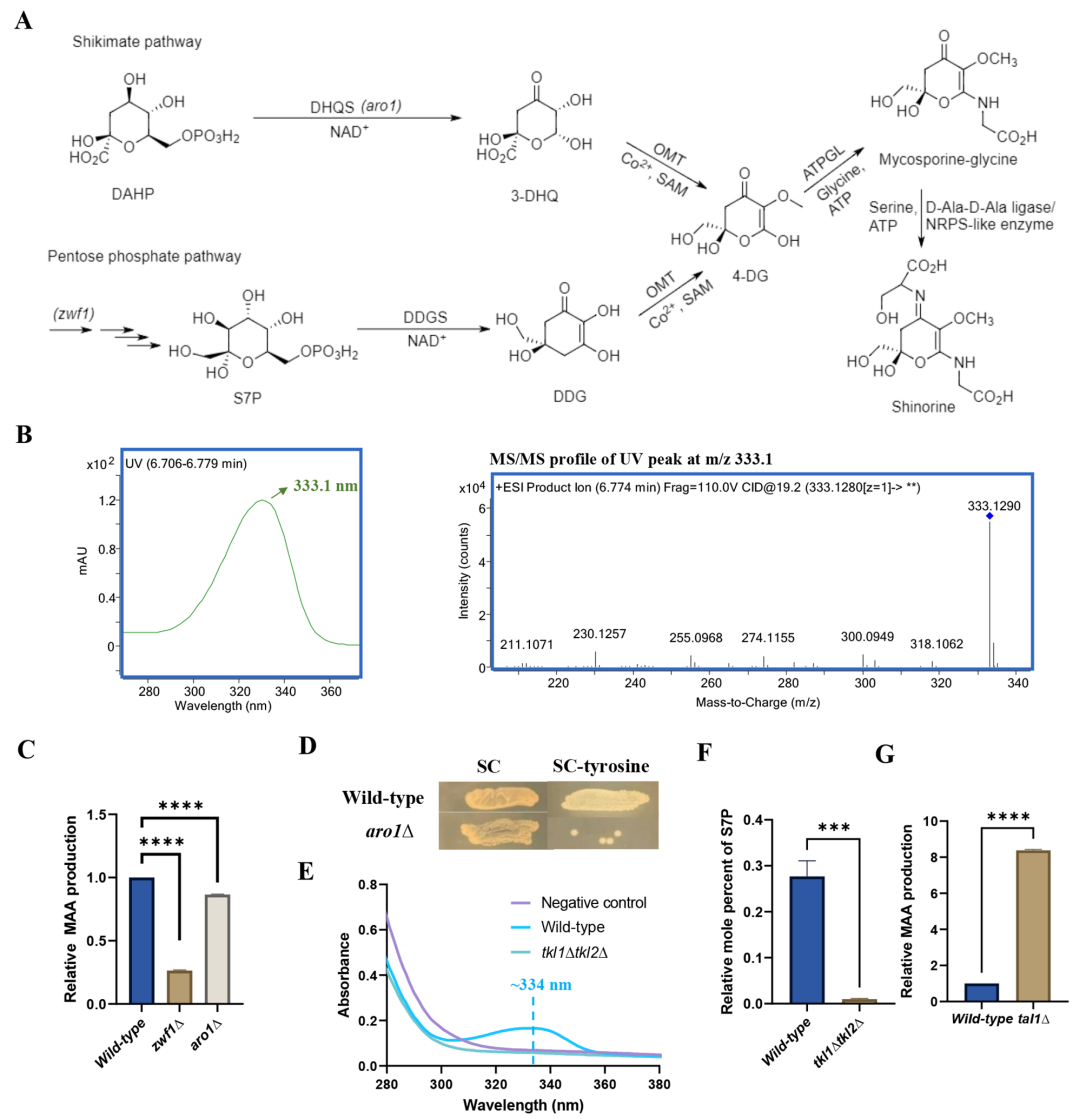
Recombinant production of MAAs in yeast requires the PPP but not the shikimate pathway. (**A**) Biosynthesis of MAA is proposed to occur from an intermediate derived from either the shikimate pathway or the pentose phosphate pathway. (DAHP = 3-deoxy-D-arabino-heptulosonate 7-phosphate, DHQS = 3-dehydroquinate synthase, 3-DHQ = 3-dehydroquinate, OMT = O-methyltransferase, DDG = demethyl 4-deoxygadusol, 4-DG = 4-deoxygadusol, ATPGL = ATP-grasp ligase, SAM = S-adenosyl methionine) (**B**) LC/MS analysis shows that the compound eluting at 6.733 min absorbs UV maximally at about 333 nm (left panel), and its corresponding MS/MS profile (right panel) agrees with the published MS/MS profile of shinorine. (**C**) Comparison of shinorine levels in the wild-type, *zwf1∆* and *aro1∆* strains. (**D**) The growth of the wild-type and *aro1∆* cells on synthetic complete (SC) and tyrosine-dropout agar plates. (**E**) The UV absorption spectra of the *tkl1∆tkl2∆* strain compared to the wild-type and negative control strains. The negative control strain refers to a strain which does not produce shinorine, as it only contains the first two genes (DDGS and OMT) of the shinorine biosynthetic pathway. (**F**) The relative mole percent of S7P in the wild-type compared to the *tkl1∆tkl2∆* strain. (**G**) Comparison of shinorine production in the wild-type and *ta11∆* strains. Error bars represent the standard deviation (SD) of three independent experiments. Significance was determined using an unpaired *t*-test. *** *p* < 0.001, **** *p* < 0.0001

Two biosynthetic routes have been suggested for MAA production [20]. While the first route involves 3-dehydroquinate (DHQ), an intermediate of the shikimate pathway, the second route involves sedoheptulose 7-phosphate (S7P), an intermediate of the pentose phosphate pathway (PPP) (Fig. 1A). Both the glycolytic pathway and the PPP contribute to the biosynthesis of 3-dehydroquinate and sedoheptulose 7-phosphate. Phosphoenolpyruvate (a glycolytic intermediate) and erythrose 4-phosphate (a PPP intermediate) are converted into deoxy-D-arabinoheptulosonate 7-phosphate (DAHP) by DAHP synthase. DAHP is converted to DHQ by DHQ synthase, which is then converted into aromatic amino acids via additional enzymes in the shikimate pathway. Sedoheptulose 7-phosphate is an intermediate in the nonoxidative branch of the PPP, and is generated from ribose 5-phosphate and xylulose 5-phosphate via the enzymatic action of transketolase. Sedoheptulose 7-phosphate is recycled to glycolytic metabolites, fructose 6-phophate and glyceraldehyde 3-phosphate, through the sequential actions of transaldolase and transketolase.

Evidence for DHQ as an MAA precursor includes the repression of shinorine synthesis in its native producers upon treatment with glyphosate, which is a shikimate pathway inhibitor [20]. On the other hand, S7P was proposed as an MAA intermediate based on the biochemical characterisation of shinorine biosynthetic gene clusters from the cyanobacteria *Nostoc punctiforme* and *Anabaena variabilis* [21]. Shinorine is synthesized from S7P over four enzymatic steps: S7P is firstly converted to 4-deoxygadusol (4-DG) by 2-demethyl 4-deoxygadusol synthase (DDGS) and O-methyltransferase (OMT) [22], followed by the conjugation of glycine to 4-DG via an ATP-grasp ligase to form mycosporine-glycine (MG) (Fig. 1A). A serine molecule is then conjugated to MG, either via a non-ribosomal peptide synthase (NRPS)-like enzyme (such as in *A. variabilis*), or by a D-Ala-D-Ala ligase (Ddl) (as in *N. punctiforme*), to produce shinorine (Fig. 1A) [21]. Shinorine production in *S. cerevisiae* through the integration of the MAA biosynthetic genes from *N. punctiforme* into the yeast genome has previously been reported [21].

In this study, we perturbed the glycolytic and pentose phosphate pathways in an engineered shinorine-producing strain of *S. cerevisiae* to increase flux towards S7P, and therefore towards MAA production. Upon finding that *PFK2* deletion enhances MAA production in yeast, we investigated the mechanism behind this boost. We show that *pfk2∆*-mediated increase in MAA production is not entirely due to increased flux towards PPP. We demonstrate that a reversal of reaction directionality between the NOPPP and glycolysis increases S7P and MAA production. This study illustrates how the NOPPP can be manipulated to enhance the production of S7P-dependent bioproducts and widens the range of metabolic engineering strategies that can be employed to increase MAA production in yeast.

## Materials and methods

### Media and culture conditions

Yeast cells were grown in yeast extract peptone (YEP) or synthetic complete (SC) medium (comprising 6.7 g/L yeast nitrogen base without amino acids and the appropriate amino acid drop-out mix), supplemented with either 20 g/L of glucose or a combination of ethanol (20 g/L) and glycerol (20 g/L) as a carbon source. For small-scale fermentation, yeast cells were grown in 10 ml SC medium with either glucose (or ethanol and glycerol) and cultured in 50 ml falcon tubes or conical flasks incubated at 30 °C with shaking (250 rpm). *Escherichia coli* Top10 strain was used for routine plasmid cloning. *E. coli* strains were aerobically grown in Luria Bertani medium at 37 °C, with antibiotic supplementation as required.

### Yeast genetic engineering

Genotypes of yeast strains used in this study are listed in Additional File 1: Table S1. *S. cerevisiae* W303 was the parental strain of all engineered strains. Plasmids and dsDNAs were transformed into yeast cells through the lithium acetate/single strand carrier DNA/polyethylene glycol method [23]. A polymerase chain reaction (PCR)-based gene deletion strategy was used to create loss-of-function deletion mutants, utilizing homologous recombination to generate a start-to-stop codon replacement of each open reading frame. The targeted ORF was replaced by a *KANMX6* or *HIS3MX6* module. To direct the amplicon towards its target site, the deletion cassette primers were designed with 5’ ends homologous (40–60 bp) to the desired integration sites. Successful transformants were selected on YPD plates supplemented with the suitable antibiotics or synthetic drop-out plates, and then confirmed by colony PCR. To construct the *pfk2∆tkl1∆tkl2∆* triple knockout strain, as well as some other knockout strains, a *MATa pfk2::KANMX6*, *tkl1::HIS3MX6* strain was crossed with a *MATα tkl2::HIS3MX6* strain, followed by sporulation and tetrad dissection to obtain the desired haploid knockout strain. Transformants of 2-micron plasmids and their derivatives expressing enzymes used in the study were selected on synthetic medium plates lacking uracil.

To integrate the shinorine biosynthetic genes into delta sites in the *S. cerevisiae* chromosome, gene expression cassettes obtained by PCR were inserted into an EK2 cloning vector pBR322 using Gibson assembly [16]. These plasmids were then treated with NotI and SalI, and the resultant DNA fragments flanked by delta sequences were transformed into yeast cells. Successful transformants were identified on YPD plates supplemented with Geneticin (G418) or Nourseothricin sulphate (clonNAT). To determine the copy numbers of the genes integrated into the genome, quantitative PCR (qPCR) was performed using gene-specific primers and SYBR Green I master mix. The *ACT1* gene was used as the endogenous control gene. The crossing point (Cp) values were determined using LightCycler Software (Roche Applied Science), and expression levels were compared to that of the reference control.

### Analysis of shinorine levels

The optical density at 600 nm (OD_600_) measured using the TECAN Infinite M200 Pro UV-visible spectrophotometer was used to determine cell growth. To estimate the quantities of shinorine produced by each strain, yeast cells from a 10 ml culture grown overnight were firstly collected by centrifugation. The cells were then resuspended in 2 ml of water, and centrifuged again to remove the supernatant. The pellet was then resuspended in 14% ethanol and vortexed, followed by incubation at 80 °C for 10 min. Following the ethanolic extraction, centrifugation was performed to obtain the supernatant, from which the amount of shinorine produced was quantified by measuring the absorbance of the supernatant between the wavelengths of 220 to 500 nm. The approximate shinorine yields of the wild-type, *pfk2Δ*, *tal1Δ* and *pfk2Δtal1Δ* strains from 10 ml overnight cultures were calculated using Beer’s law, with the molar extinction coefficient of shinorine taken to be ε = 44 668 M^− 1^ cm^− 1^ [24]. These approximate yields are reported in Additional File 1: Figure S1.

The ratio of absorbance at 334 nm (maximal absorption wavelength of shinorine) to the absorbance at 260 nm ($\frac{{A}_{334\ nm}}{{A}_{260\ nm}}$) of the ethanolic extracts from each yeast strain was taken to reflect its shinorine levels, normalized to the number of cells in each sample (measured by the amount of DNA/RNA). This value was then subtracted by the $\frac{{A}_{334\ nm}}{{A}_{260\ nm}}$ value of the negative control (a yeast strain containing the genes DDGS and OMT only) to eliminate non-specific background readings. The relative amount of shinorine production compared to the wild-type strain was then determined by taking the ratio of $({\frac{{A}_{334\ nm}}{{A}_{260\ nm}})}_{normalized\ sample}$ to $({\frac{{A}_{334\ nm}}{{A}_{260\ nm}})}_{normalized\ wild-type}$

The overall formula used was therefore:

$$Relative\ MAA\ production =\frac{({\frac{{A}_{334\ nm}}{{A}_{260\ nm}})}_{sample}-{\left(\frac{{A}_{334\ nm}}{{A}_{260\ nm}}\right)}_{negative\ control}}{({\frac{{A}_{334\ nm}}{{A}_{260\ nm}})}_{wild-type}-{\left(\frac{{A}_{334\ nm}}{{A}_{260\ nm}}\right)}_{negative\ control}}$$

### Confirmation of shinorine production

Lyophilized cell pellet was extracted using a 50% methanol solution and sonicated in an ice-water bath for 30 min. The extract was centrifuged, and supernatant was transferred to an autosampler vial. 5 uL of extract was injected for liquid chromatography-tandem mass spectrometry (LC-MS/MS) analysis. The sample was run on an Agilent 1290 Ultra-High Performance Liquid Chromatography coupled to an Agilent 1290 Infinity Diode Array detector and an Agilent 6540 UHD quadrupole time-of-flight (Q-TOF) mass spectrometer. Separation was done on a SeQuant® ZIC®-HILIC column using gradient elution with mobile phases comprising 0.1% formic acid in both acetonitrile and water; flow rate at 0.3 mL/min. MS2 data was acquired in data dependent Auto MS/MS acquisition mode under positive electrospray ionization conditions. The typical Q-TOF operating parameters were as follows: sheath gas nitrogen flow, 12 L/min at 230 °C; drying gas nitrogen flow, 8 L/min at 300 °C; nebulizer pressure, 30 psi; nozzle voltage, 1.5 kV; capillary voltage, 4 kV. Lock masses in positive ion mode: purine at m/z 121.0509 and HP-0921 at m/z 922.0098.

### Analysis of intracellular metabolites

Glycolytic and pentose phosphate pathway metabolites were extracted from stationary phase cells. Overnight cultures were grown in synthetic complete media, quenched by addition to twice the volume of methanol (preincubated at -80 °C) and immediately pelleted at 4000 g in a pre-cooled centrifuge (4 °C) for 2 min. Supernatants were discarded and pellets were processed as previously described [25, 26].

### ^**13**^**C-labelling**

Yeast strains were grown in synthetic complete media with 2% unlabelled glucose until they reached log-phase. 0.5% [1-^13^C]-glucose (Catalog No. 297046, Merck) was then added to the medium, and after 10 min, the strains were quenched with twice the volume of methanol (preincubated at -80 °C). The cells were immediately pelleted at 4000 g in a pre-cooled centrifuge (4 °C) for 2 min. Metabolite analysis was subsequently carried out as described above. The area of the labelled S7P detected was adjusted by the proportion of natural ^13^C isotope present, and the ratio of peak areas between labelled and unlabelled S7P was calculated to demonstrate the relative proportion of glucose that is directed towards glycolysis over the oxidative pentose phosphate pathway.

### Enzyme constrained flux balance analysis

Enzyme constrained flux balance analysis (FBA) was carried out following previous work [27, 28], to analyze the metabolic phenotype of yeast cells under various conditions. Briefly, enzyme constrained FBA is a constraint-based modelling approach which considers that enzymes catalyzing reactions in a cellular system compete for the available cytoplasmic state. Considering the very high intracellular concentration of macromolecules [29, 30], there exists physical and spatial constraints that limit enzyme levels within a given cell volume *V*, such that:

$$\sum _{i=1}^{N}{v}_{i}{n}_{i}\le V$$

where ${v}_{i}$ is the molar volume of the *i*th enzyme and ${n}_{i}$ is the number of moles of the *i*th enzyme. In terms of enzyme concentrations ${E}_{i}=\frac{{n}_{i}}{M}$, measured in moles per unit mass, this constraint then becomes:

$$\sum _{i=1}^{N}{v}_{i}{E}_{i}\le \frac{1}{C}$$

where $C=\frac{M}{V}\approx$ 0.3 g/ml is the cytoplasmic density of the *S. cerevisiae* cell [28, 31]. The Yeast8 model [32] was therefore constrained by the constant C to reflect this enzyme concentration constraint in the cell.

The overall enzyme capacity was constrained by incorporating stoichiometric coefficient values based on the MW/k_cat_ of corresponding enzymes in a pseudo-reaction, where MW refers to the molecular weight of the enzyme corresponding to the reaction and k_cat_ is the turnover number of the same enzyme. MW and k_cat_ values were extracted from a previous report [33]. Where the MW or k_cat_ data was not available for a particular reaction, random values were assigned from the permuted list of known values; 5000 different permutations were used to obtain 5000 flux solutions for each simulation, and the mean of the resultant flux solutions was used to represent the average cellular state for each condition. A sink reaction to reflect the accumulation of S7P was added to the model. Experimental observations upon the partial deletion of phosphofructokinase activity in the cell were incorporated in the *PFK*-knockout model through the additional constraint of flux in the forward reaction of *PGI1*, and the limitation of flux through *ZWF1* to approximately half of that in the wild-type. The model used and the fluxes calculated can be found in Additional File 2.

## Results

### A system for studying factors that regulate MAA production in yeast

To identify bottlenecks limiting production of MAAs in *S. cerevisiae*, we first constructed a shinorine-producing yeast strain. The four shinorine biosynthetic genes from cyanobacterium *N. punctiforme*, which encode the enzymes DDGS (*NpR5600*), OMT (*NpR5599*), ATP-grasp ligase (ATPGL) (*NpR5598*) and D-Ala-D-Ala ligase (DADA) (*NpR5597*) were expressed in yeast. We split the four biosynthetic genes into two expression modules, with Module 1 consisting of DDGS and OMT, and Module 2 consisting of ATPGL/DADA. To obtain high production of shinorine, we integrated the two modules at the delta element sequences, which are long terminal repeats (LTR) flanking retrotransposons Ty1 and Ty2. The *S. cerevisiae* genome contains hundreds of delta element sequences [34], enabling the multicopy integration of target genes through delta sites-mediated homologous recombination. To identify a promoter combination that maximizes shinorine production, we expressed the two modules under three different promoters, namely P_*TEF1*_, P_*TPI1*_, or P_*ADH1*_, and generated 6 different strains. The strain in which both modules are under the control of the *TEF1* promoter resulted in the highest shinorine production. The identity of shinorine was confirmed by LC-MS/MS. The extracted ion chromatogram (EIC) of shinorine C_13_H_20_N_2_O_8_, target mass m/z 333.1298 [M + H]^+^, revealed a peak at retention time (RT) 6.83 min, which corresponded to the major peak at RT 6.73 min observed at UV 333 nm (Additional File 1: Figure S2). The UV spectrum of the major peak exhibited a λ_max_ at 333 nm and the MS/MS spectra of m/z 333.1298 (Fig. 1B) were in good agreement with that of shinorine [8]. To readily test the effect of genetic changes on shinorine production, we integrated the two modules driven by P_*TEF1*_ into the wild-type haploid strain to construct the strain YMAA1.

### Blocking the pentose phosphate pathway severely impacts shinorine production

Biosynthesis of MAAs has been proposed to occur from either sedoheptulose 7-phosphate (S7P), derived from the pentose phosphate pathway (PPP), or 3-dehydroquinate (DHQ) derived from the shikimate pathway (Fig. 1A) [20]. To distinguish between the two possibilities, we tested the effect of blocking either the PPP or the shikimate pathway on shinorine production, by deleting the *ZWF1* and *ARO1* genes respectively from the strain YMAA1. *ZWF1* encodes the glucose 6-phosphate dehydrogenase catalyzing the first committed step of the PPP, while *ARO1* encodes the penta-functional enzyme that converts 7-phospho-2-dehydro-3-deoxy-D-arabino-heptonate to 3-dehydroquinate. *zwf1∆* decreased shinorine production by approximately 74% (Fig. 1C). In contrast, *aro1∆* had a far smaller effect (16% reduction) on shinorine production. We confirmed that the *aro1∆* mutant was unable to grow in the absence of tyrosine (Fig. 1D). This indicates that shinorine production is not dependent on DHQ from the shikimate pathway. While *zwf1∆* had a significant effect, it did not totally block shinorine production. To assess whether shikimate pathway contributes to shinorine production in the *zwf1∆* mutant, we constructed the *zwf1*∆*aro1*∆ mutant. Shinorine production in the *zwf1*∆*aro1*∆ and *zwf1Δ* mutants were comparable (Additional File 1: Figure S3), indicating that shikimate pathway is dispensable for shinorine production.

### Transketolases are essential for shinorine production

As the *zwf1∆* strain is viable, we reasoned that essential pentose phosphates such as ribose 5-phosphate, ribulose 5-phosphate and xylulose 5-phosphate must be produced via other biosynthetic pathways; these pentose phosphates can then be converted to S7P by transketolases. To test this possibility, we deleted the *TKL1* and *TKL2* genes, which encode the two transketolases in yeast [35]. While *tkl1Δ* reduced shinorine production levels by 57% compared to the wild-type, *tkl2Δ* had a more modest effect (9% reduction) (Additional File 1: Figure S4). However, the *tkl1Δtkl2Δ* mutant did not produce any shinorine (Fig. 1E/F), indicating that transketolase activity is essential for shinorine production in yeast. Metabolite analyses also revealed that S7P levels were highly depleted in the *tkl1Δtkl2Δ* strain compared to the wild-type strain, strongly suggesting that shinorine is produced from S7P in yeast.

### **Deletion of*TAL1*boosts shinorine production**

Transaldolase transfers the dihydroxyacetone phosphate from S7P into glyceraldehyde 3-phosphate (G3P) to form erythrose 4-phosphate (E4P) and fructose 6-phosphate (F6P). Blocking transaldolase activity should therefore boost S7P levels. Indeed, deletion of *TAL1* has been shown to lead to accumulation of S7P [36], and increased shinorine production in yeast [16]. We also found that the *tal1Δ* mutant strain produced 9-fold more shinorine compared to the wild-type strain (Fig. 1G). *NQM1* encodes a transaldolase and is a paralog of *TAL1*. Unlike *tal1∆*, deleting *NQM1* had no effect on shinorine levels (Additional File 1: Figure S5). Moreover, shinorine levels in *tal1∆nqm1∆* and *tal1∆* strains were comparable, indicating that *NQM1* does not regulate S7P levels in yeast. Analyses involving knockouts of other genes involved in the metabolism of S7P in *S. cerevisiae*, such as *SHB17*, which hydrolyzes sedoheptulose 1,7-bisphosphate (SBP) to S7P, and *PHO13*, which dephosphorylates S7P to sedoheptulose [37], were also carried out. However, these were similarly found to have an insignificant impact on shinorine production (Additional File 1: Figure S6).

### Reducing phosphofructokinase activity boosts shinorine production

**Fig. 2:**
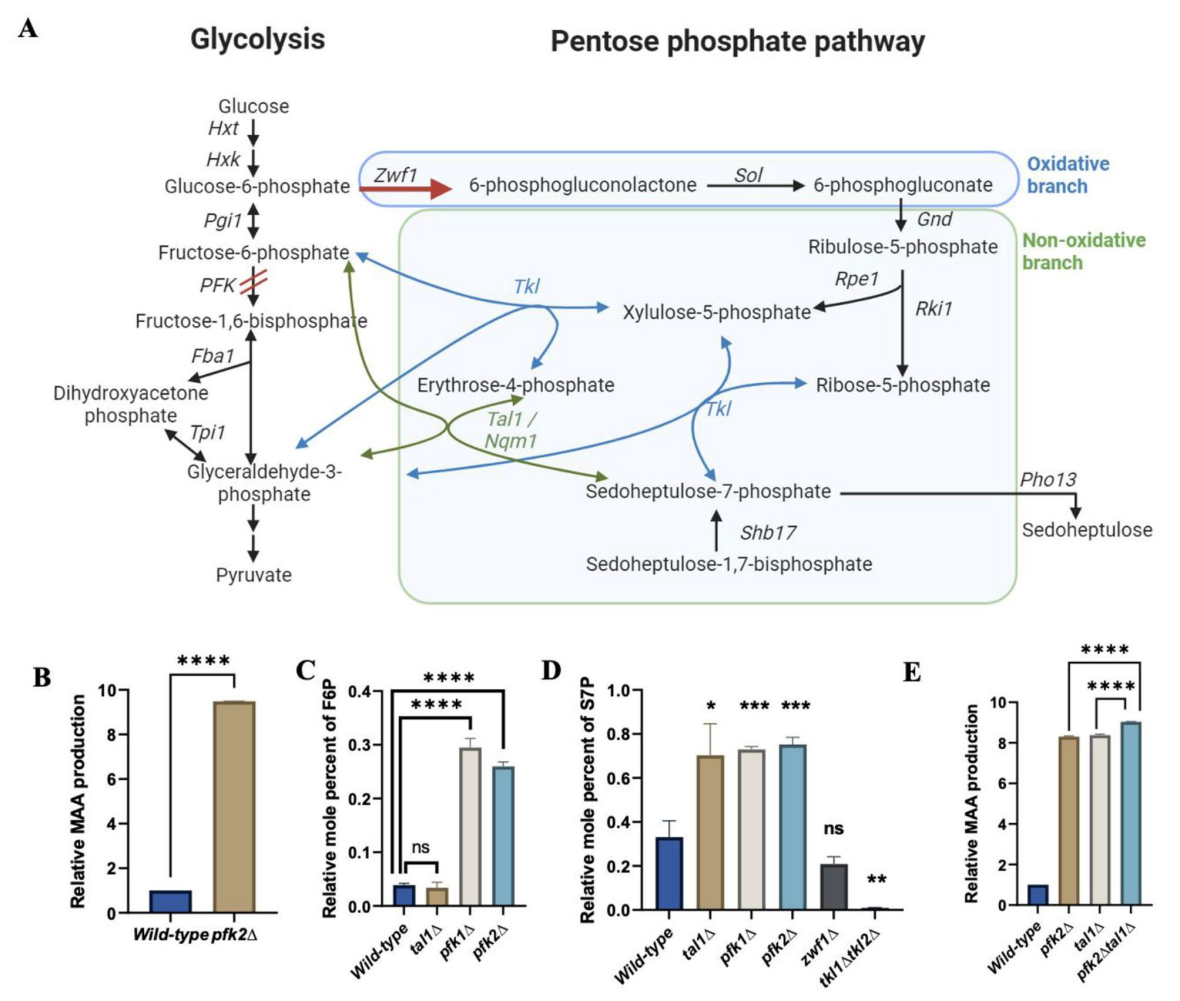
*tal1∆* and *pfk2∆* enhance shinorine production by independent mechanisms in yeast. (**A**) A schematic showing the glycolytic pathway, the pentose phosphate pathway and other reactions related to sedoheptulose 7-phosphate metabolism in *S. cerevisiae.* Blocking the phosphofructokinase reaction would theoretically increase flux through the oxidative branch of the PPP via *ZWF1* (as indicated in red). (**B**) Comparison of shinorine levels in the wild-type and *pfk2∆* strains. (**C**) The levels of fructose 6-phosphate produced in wild-type, *tal1∆, pfk1∆*, and *pfk2∆* strains. (**D**) The effect of *tal1∆, pfk1∆, pfk2∆, zwf1∆* and *pfk1∆pfk2∆* on levels of S7P present in the cell, which correspond to the levels of shinorine produced. (**E**) Comparison of shinorine levels in the wild-type, *pfk2∆, tal1∆* and *pfk2∆tal1∆* strains. Error bars represent the standard deviation (SD) of three independent experiments. Significance was determined using an unpaired *t*-test. In Fig. 2D, the asterisks above the sample bars denote significance relative to the wild-type strain. ns = not significant, * *p* < 0.05, ** *p* < 0.01, *** *p* < 0.001, **** *p* < 0.0001

As flux towards PPP is low in comparison to glycolytic pathway in *S. cerevisiae* [38], we hypothesized that reducing glycolytic flux would enhance flux towards PPP and thereby boost S7P and shinorine production. Phosphofructokinase in yeast is a heterooctameric complex composed of Pfk1 and Pfk2 tetramers and phosphorylates fructose 6-phosphate to produce fructose 1,6-bisphosphate (Fig. 2A). Inactivation of both Pfk1 and Pfk2 subunits impairs growth of yeast cells in glucose (Additional File 1: Figure S7), but the single *PFK* mutants can grow in glucose albeit at reduced growth rates [17]. To reduce glycolytic flux, we deleted the *PFK2* gene and examined its effect on shinorine production. *pfk2Δ* boosted shinorine levels by about 9-fold compared to wild-type cells (Fig. 2B). Transformation of the shinorine-producing *pfk2Δ* strain with wild-type and catalytically-impaired versions of the *S. cerevisiae* Pfk2 revealed that the loss of *PFK’s* catalytic activity is required for the observed boost in shinorine production (Additional File 1: Figure S8).

To test if deletion of phosphofructokinase genes boosts shinorine production by increasing S7P levels, we performed targeted metabolite analysis of wild-type and *pfk1∆/pfk2∆* cells by mass spectrometry. We used the *tal1∆*, *tkl1∆tkl2∆* and *zwf1∆* strains as controls. More F6P accumulation was observed in the *pfk1∆* and *pfk2*∆ mutants compared to the wild-type cells (Fig. 2C). The *tkl1∆tkl2∆* strains produced no S7P, while *zwf1∆* decreased the relative mole percent of S7P among the PPP metabolites by 37%. Importantly, the proportion of intracellular S7P among the glycolytic metabolites produced by the *tal1∆* strain increased by about two-fold from 31 to 63%; similarly, the *pfk2∆* mutant demonstrated significantly heightened levels of S7P (Fig. 2D).

Deletion of *PFK2* and *TAL1* genes is expected to boost S7P levels via distinct mechanisms, since the deletion of *TAL1* accumulates S7P by blocking the transaldolase reaction, and the deletion of *PFK2* is posited to redirect glycolytic flux towards the PPP. Indeed, shinorine production in the *pfk2Δtal1Δ* strain was more than in the corresponding single mutants (Fig. 2E), suggesting independent mechanisms which have a cumulative effect on S7P/shinorine production.

### **Enhanced shinorine production in the*pfk2∆*mutant is not due to increased PPP flux**

**Fig. 3:**
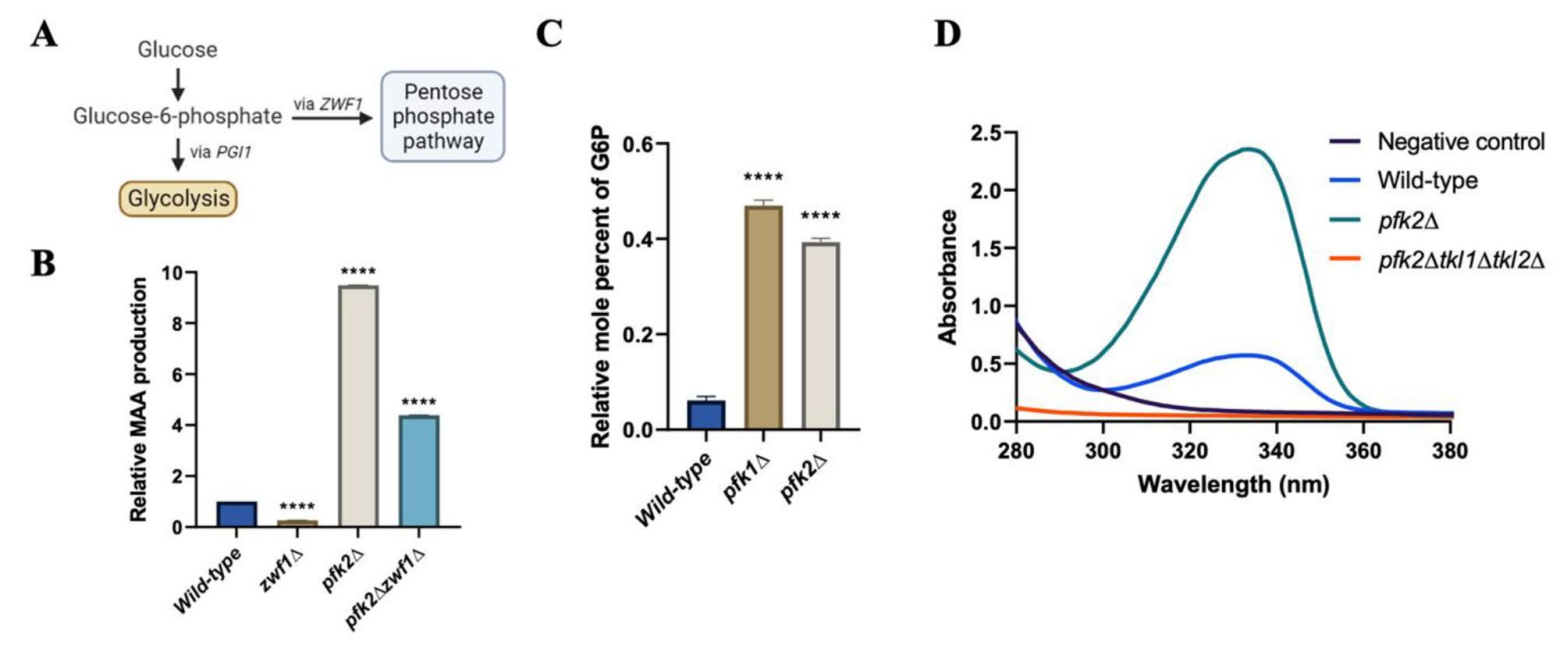
The *pfk2∆*-mediated boost in MAA production is not due to increased flux via PPP. (**A**) Glucose 6-phosphate obtained from hexokinase-mediated phosphorylation of glucose can be metabolized either via the glycolytic pathway through *PGI1*, or via the pentose phosphate pathway through *ZWF1*. (**B**) Comparison of shinorine levels in the wild-type, *zwf1∆, pfk2∆* and *pfk2∆zwf1∆* strains. (**C**) Relative levels of glucose 6-phosphate in the wild-type, *pfk1∆* and *pfk2∆* cells. Error bars represent the standard deviation (SD) of three independent experiments. Significance was determined using an unpaired *t*-test. The asterisks above the sample bars denote significance relative to the wild-type strain. **** *p* < 0.0001. (**D**) Comparison of MAA levels in the negative control, wild-type, *pfk2∆* and *pfk2∆tkl1∆tkl2∆* strains

In the metabolic network towards the production of S7P in *S. cerevisiae*, glucose 6-phosphate can either enter the oxidative PPP via *ZWF1* or proceed through the glycolytic pathway via *PGI1* and *PFK1/2* (Fig. 3A). To determine if the increase in S7P/MAA levels in the *pfk2Δ* strain is due to increased flux through the PPP, we tested the effect of deleting *ZWF1* on shinorine production in the *pfk2∆* strain. Surprisingly, the *pfk2Δzwf1Δ* mutant still showed a significant boost in MAA production compared to the wild-type and the *zwf1Δ* single mutant (Fig. 3B), suggesting that the *pfk2Δ*-mediated boost is not solely attributable to increased flux towards the PPP. This is despite the significant accumulation of glucose 6-phosphate observed in the *pfk1Δ* and *pfk2Δ* strains (Fig. 3C), which thermodynamically would increase flux through the PPP reactions. It is formally possible that deletion of *PFK2* triggers the activation of a previously unknown pathway for S7P production distinct from either glycolysis or the PPP. However, deletion of *TKL1* and *TKL2* genes completely blocked MAA production in the *pfk2Δ* strain, ruling out this possibility (Fig. 3D).

### ***pfk2Δ*-mediated boost in MAA production is not due to the activation of the ribose salvage pathway**

Carbon starvation causes accumulation of S7P in yeast cells via the ribose salvage pathway by the action of autophagic enzymes such as *ATG7*, and nucleoside phosphorylases (e.g. *PNP1*) and hydrolases (*URH1*) (Additional File 1: Figure S9A) [39]. Since *PFK2* deletion could potentially mimic carbon starvation by reducing glycolytic flux, S7P accumulation in *pfk2Δ* mutant could be attributable to this pathway. However, *atg7Δ*, *pnp1Δ*, *urh1Δ*, and *prm15Δ* did not have a significant effect on shinorine production in the *pfk2Δ* strain (Additional File 1: Figure S9B), ruling out this possibility.

### Reversed non-oxidative PPP

**Fig. 4:**
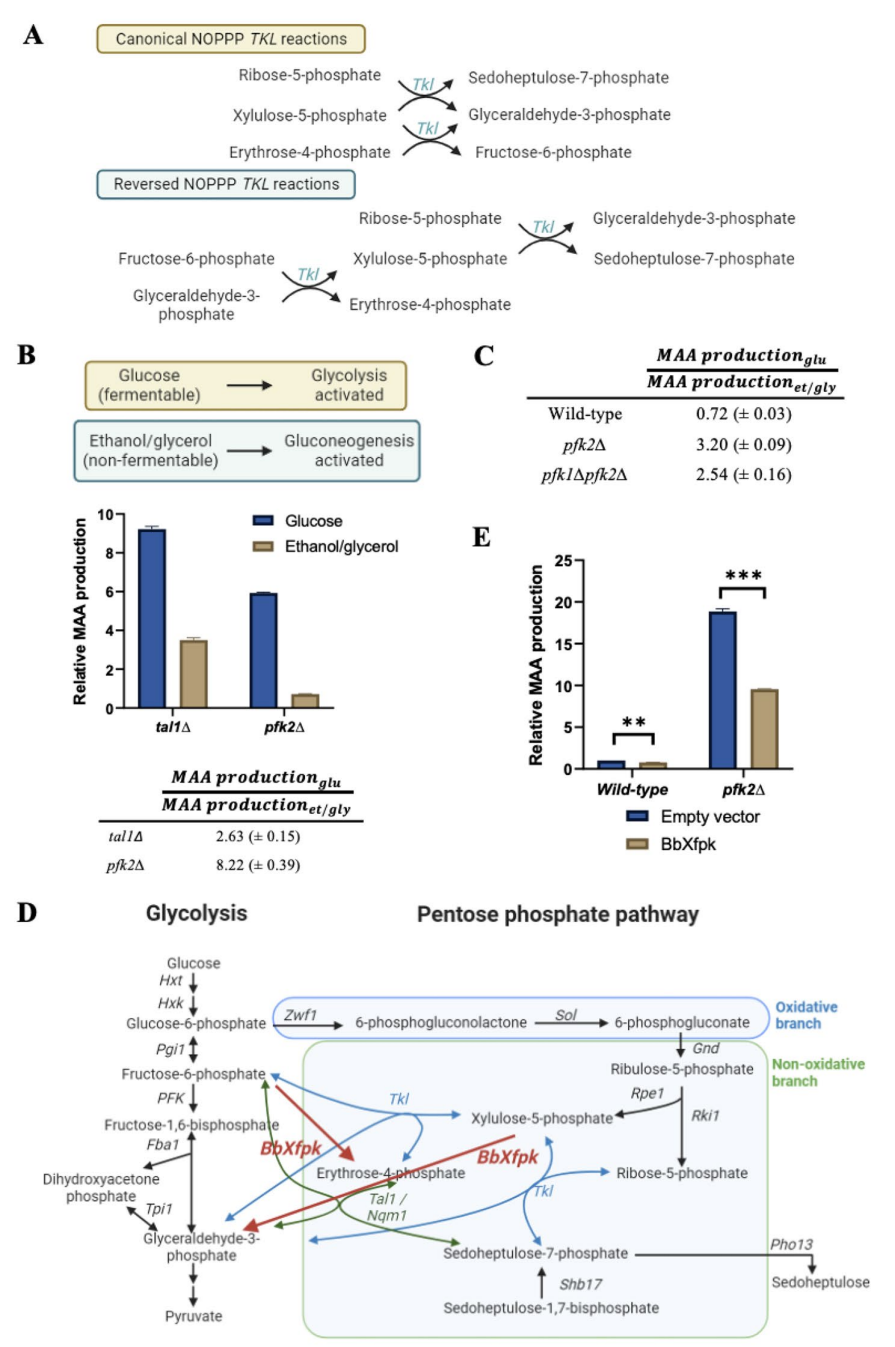
Testing the Reversed NOPPP model for the *pfk2∆*-mediated boost in shinorine production. (**A**) An illustration of the transketolase-catalyzed reactions in the canonical non-oxidative PPP, and in the reversed non-oxidative PPP, where the direction of the *TKL*-mediated reaction involving F6P and G3P is reversed. (**B**) While *S. cerevisiae* cells grown in fermentable carbon sources undergo glycolysis, the cells grown in non-fermentable carbon sources undergo gluconeogenesis. The levels of shinorine production in the *pfk2Δ* and *tal1Δ* strains grown in SC-glucose and SC-ethanol/glycerol. (**C**) Shinorine production in the wild-type, *pfk2Δ*, and *pfk1Δpfk2Δ* strains after overnight growth in SC-ethanol/glycerol, and upon addition of 2% glucose to the overnight cultures. (**D**) Reactions catalyzed by BbXfpk in the context of the yeast glycolytic and pentose phosphate pathways. (**E**) The effect of overexpressing an F6P-consuming bacterial phosphoketolase on shinorine production in the wild-type and *pfk2Δ* strains. Error bars represent the standard deviation (SD) of three independent experiments. Significance was determined using an unpaired *t*-test. ** *p* < 0.01, *** *p* < 0.001

As the boost in shinorine production in the *pfk2∆* mutant is not solely attributable to an increased flux through the PPP or the activation of the ribose salvage pathway, we explored alternative scenarios. Other than the previously reported idea that glycolytic suppression increases flux through the oxidative PPP via G6P accumulation [40, 41], we envisaged a possible parallel pathway in which the fructose 6-phosphate, which accumulates in the *pfk2∆* mutant, gets converted into S7P via the non-oxidative PPP, but with one of the transketolase reactions occurring in the reverse direction (Fig. 4A). We refer to this as Reversed NOPPP for the remainder of this manuscript.

### **Growth of yeast cells in a fermentable carbon source is required for the*pfk2∆*-mediated boost in shinorine production**

Production of F6P from G6P requires the action of the glycolytic enzyme phosphoglucoisomerase. Glycolysis is inactive in yeast cells growing in a non-fermentable carbon source [42]. If the Reversed NOPPP hypothesis were true, then growth of *pfk2∆* cells in a non-fermentable carbon source should abolish the increase in shinorine production. We cultured wild-type, *pfk2∆* and *tal1∆* cells in glucose or a non-fermentable carbon source (2% ethanol and glycerol) and compared their relative shinorine production. The *pfk2Δ* strain produced about 8-fold more shinorine when grown in glucose when compared to the non-fermentable carbon source (Fig. 4B). On the other hand, shinorine production in the *tal1Δ* strain was just 2.63-fold more in glucose compared to the non-fermentable carbon source. These results indicate that shinorine overproduction in the *pfk2Δ* strain requires the glycolytic pathway to be active, which is consistent with the Reversed NOPPP hypothesis.

The Reversed NOPPP hypothesis posits that glycolytic steps downstream of *PFK* are not critical for the *pfk2∆*-mediated boost in shinorine production (Fig. 4A). Therefore, abolishing phosphofructokinase activity completely should continue to boost shinorine production. To test this, we grew wild-type, *pfk1∆*, *pfk2∆* and *pfk1∆pfk2∆* cells in ethanol/glycerol to mid-log phase and assessed their shinorine production following addition of 2% glucose to the cultures. Addition of glucose to the wild-type cells did not have much of an effect on shinorine levels. However, shinorine levels increased in the *pfk2∆* and *pfk1∆pfk2∆* mutants by 3.20- and 2.54-fold respectively (Fig. 4C). While the downstream glycolytic metabolite G3P is also essential for shinorine production through the Reversed NOPPP, yeast cells grown in ethanol/glycerol are expected to contain high levels of G3P from gluconeogenesis [43, 44]. These results suggest that accumulation of F6P upon phosphofructokinase inactivation is important for the increase in shinorine production.

### **Expression of phosphoketolases reduces the** ***pfk2Δ*-mediated boost in shinorine production**

If F6P levels are important for the boost in shinorine production in the *pfk2∆* mutant, then depleting F6P levels should reduce shinorine production. To test this possibility, we overexpressed a phosphoketolase from *Brevibacillus breve* (BbXfpk) (Fig. 4D), which catalyzes the following reactions: [45]

$$F6P \to E4P{\rm{ }} + {\rm{ }}AcP$$

$$X5P \to G3P{\rm{ }} + {\rm{ }}AcP$$

BbXfpk has a high capability of converting fructose 6-phosphate to erythrose 4-phosphate and acyl phosphate, and its expression has successfully been used to divert glycolytic flux towards E4P in *S. cerevisiae* [46]. We drove BbXfpk expression in the shinorine-producing strain using the strong constitutive promoter *ADH1* in a multi-copy 2-micron plasmid. Overexpression of BbXfpk caused shinorine production in the *pfk2Δ* strain to decrease by approximately 49%, compared to the much smaller 12% decrease in the wild-type strain (Fig. 4E). These results further support the importance of F6P accumulation for the shinorine production boost in the *pfk2∆* mutant.

### ^**13**^**C-labelling in*pfk2∆*reveals increased flux through the Reversed NOPPP**

**Fig. 5:**
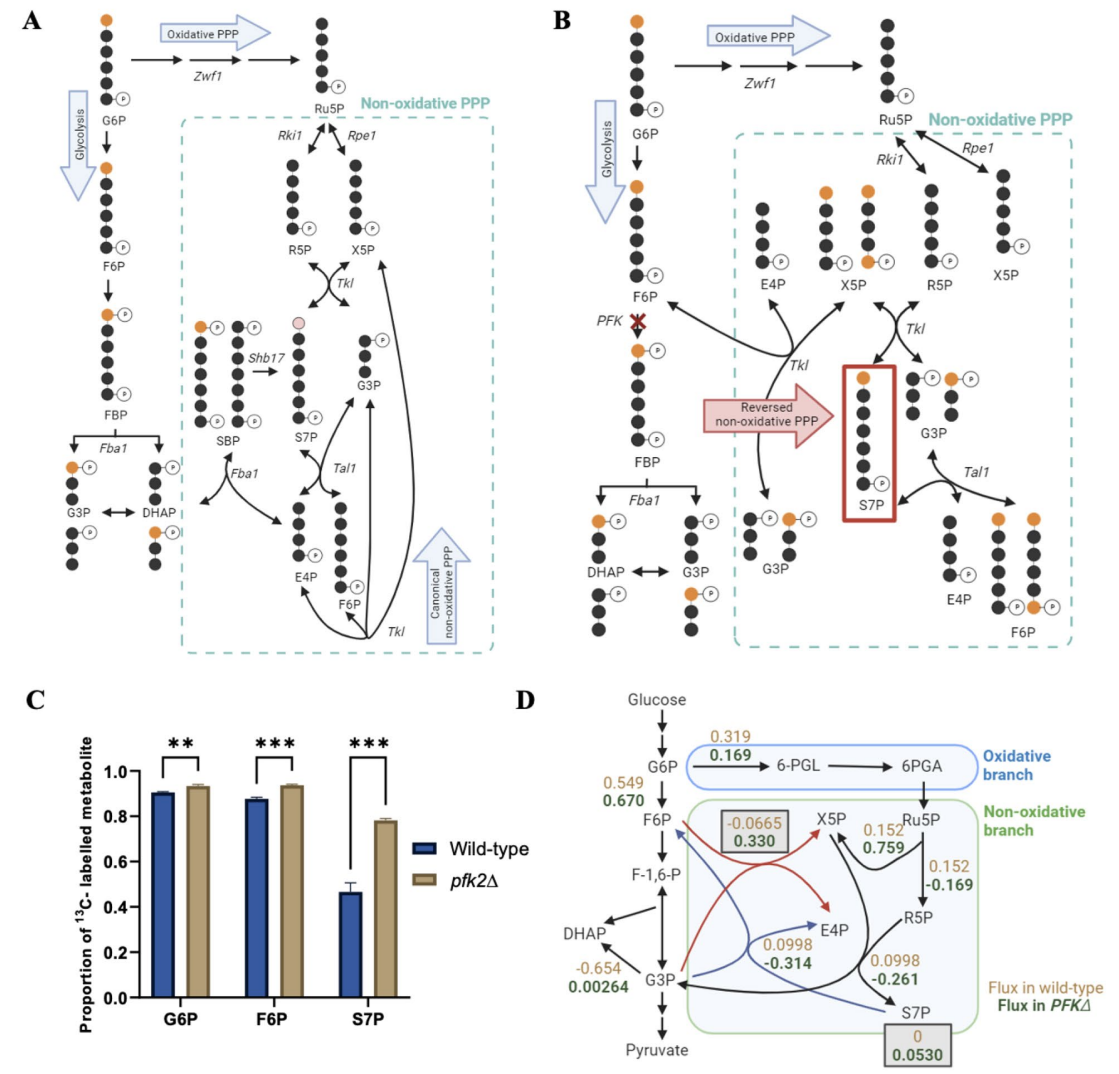
Metabolite labelling experiments and flux balance analyses support the Reversed NOPPP model. (**A**) [1-^13^C]-glucose metabolism leads to unlabelled S7P when S7P is made via the PPP alone, as the first carbon is lost as carbon dioxide in the oxidative PPP. A small proportion of [1-^13^C]-S7P could be detected through the S7P-producing reaction of Shb17 (pink). (**B**) Flux through glycolysis to S7P via the Reversed NOPPP reaction can be measured using [1-^13^C]-glucose. [1-^13^C]-glucose leads to [1-^13^C]-S7P when S7P is produced via the glycolytic and Reversed NOPPP routes. (**C**) The proportion of [1-^13^C]-G6P, [1-^13^C]-F6P and [1-^13^C]-S7P of the total amount of each metabolite detected is shown, where m/z 259 -> 199, m/z 259 -> 169 and m/z 289 -> 97 were analyzed respectively. The fragmentation patterns used for these compounds can be found in Additional File 1: Figure S10. The carbon flux through the glycolytic/Reversed NOPPP route is calculated based on the proportion of labelled [1-^13^C]-S7P among the total amount of S7P detected. Error bars represent the standard deviation (SD) of three independent experiments. Significance was determined using an unpaired *t*-test. ** *p* < 0.01, *** *p* < 0.001. (**D**) The effect of *PFK* deletion on fluxes through key glycolytic and PPP reactions, as demonstrated in an enzyme-constrained model. The wild-type and *PFK-*knockout flux values are depicted in brown and dark green respectively

S7P can be produced either through the canonical pentose phosphate pathway via *ZWF1*, or through the Reversed NOPPP reaction, where transketolase converts F6P and G3P to direct carbon flux towards NOPPP metabolites. The flux through either of these reactions can be measured based on the proportion of labelled [1-^13^C]-S7P in cells fed by glucose that is singly-labelled at the C1 position, [1-^13^C]-glucose. As the C1 carbon of glucose is lost as carbon dioxide in the oxidative PPP, the metabolism of [1-^13^C]-glucose via the oxidative PPP will produce unlabelled ribulose 5-phosphate, ribose 5-phosphate, and xylulose 5-phosphate. These are subsequently metabolized to produce unlabelled S7P and G3P by transketolases (Fig. 5A). In the wild-type strain, NOPPP flux from the glycolytic intermediates primarily occurs through the dephosphorylation of SBP to form S7P, but the proportion of S7P produced through this reaction is likely to be low [37]. Moreover, only half of the S7P produced through this pathway will be [1-^13^C]-labelled. Nevertheless, we expect to see some [1-^13^C]-S7P in the wild-type strain, as transketolases are known to scramble carbon atoms through NOPPP metabolites [37].

However, when carbon flux is redirected from glycolytic intermediates to the NOPPP in the *pfk2∆* strain, S7P produced through this pathway will be [1-^13^C]-labelled. Transketolase transfers the C1 and C2 carbons of F6P to G3P, producing unlabelled E4P and either [1-^13^C]-X5P or [1,5-^13^C]-X5P. The C1 and C2 carbons of X5P are then transferred to unlabelled R5P from the oxidative PPP, producing labelled [1-^13^C]-S7P (Fig. 5B). The amount of [1-^13^C]-S7P among the total amount of S7P detected can therefore be used to reflect the relative proportion of S7P produced via glycolytic intermediates. To determine the flux through the Reversed NOPPP by this approach, wild-type and *pfk2∆* cells in first grown in SC medium supplemented with unlabelled glucose until mid-log phase and then switched to [1-^13^C]-glucose for 10 min. This labelling duration was selected to allow for the labelling to occur, while minimizing the carbon scrambling that occurs when NOPPP metabolites undergo further reactions through *TAL* and *TKL* [37].

Comparing the wild-type and *pfk2∆* strain labelling profiles, 46% of S7P in the wild-type cells were singly-labelled, while this proportion increases to 78% in the *pfk2∆* strain (Fig. 5C, Additional File 1: Figure S10). This is compared to the much more modest 3 to 5% difference in proportion of singly-labelled G6P and F6P between the wild-type and *pfk2∆* strains, as is expected with these early glycolytic metabolites. The relatively elevated proportion of singly-labelled S7P in the wild-type cells could be attributed to *TKL*/*TAL*-mediated carbon scrambling. Nevertheless, the significantly higher proportion of labelled S7P in the *pfk2∆* strain implies that when flux through the phosphofructokinase reaction is limited, majority of the carbon flux is directed through glycolysis, where F6P and G3P are converted to E4P and X5P. This therefore serves as further proof of the reverse of the canonical transketolase reaction, activated by accumulation of F6P.

### Enzyme capacity constrained flux balance analysis

We then tested whether it is possible to recapitulate our experimental results using constraint-based modeling. Genome-scale metabolic models (GEMs) contain annotated gene-protein-reaction (GPR) relationships, and all the reactions are mass- and energy-balanced to ensure stoichiometric balance. They therefore allow for system-level flux simulations and analysis of metabolic responses to be carried out, and have been used widely to guide the metabolic engineering of industrial microorganisms, including *S. cerevisiae* [47]. We established an enzyme constrained flux balance analysis (FBA) model of yeast using the Yeast8 metabolic model of *S. cerevisiae*, and compared the flux through key glycolytic and PPP reactions in the wild-type and *PFK*∆ mutant models. The *PFK*∆ mutant model was constrained by the oxidative PPP flux measurement from the carbon isotope labelling experiment. The predicted relative internal flux distributions in the two strains were highly consistent with our experimental data and the Reversed NOPPP hypothesis. The *PFK*∆ mutant demonstrates slightly increased flux through the *PGI1* reaction, a reversal of the canonical *TKL* reaction such that F6P and G3P are now converted to X5P and E4P, and an accumulation of S7P (Fig. 5D). This serves as further validation of the role of the Reversed NOPPP in S7P accumulation observed in the *PFK*∆ mutant strain, which results in the MAA accumulation demonstrated in earlier sections of the study. Additionally, the model suggests flux redirection towards the pentose phosphates X5P and R5P in the *pfk2∆* strain, possibly to meet the cellular demand for ribose which is not fully met by the oxidative PPP (i.e. where the cell is in a state of pentose insufficiency [48]).

## Discussion

To understand factors regulating MAA production in *S. cerevisiae*, we expressed the 4 shinorine biosynthetic genes from *N. punctiforme* to construct a shinorine-producing yeast strain. MAAs were hypothesized to be generated from an intermediate either from the shikimate pathway or the pentose phosphate pathway. Using genetics, we demonstrate that MAAs are produced primarily from the PPP intermediate S7P, and not from the shikimate pathway intermediate DHQ.

To improve shinorine production in *S. cerevisiae*, we redirected the metabolic flux to increase the availability of S7P. Firstly, ablation of transaldolase activity boosted MAA production by 9-fold, presumably by preventing the breakdown of S7P. This is consistent with results reported in *E. coli* and *S. cerevisiae* ( [15, 18]). Secondly, we boosted MAA production by 9-fold by deleting the phosphofructokinase gene *PFK2*. Combining the *TAL1* and *PFK2* knockouts demonstrated an additive effect on shinorine production, indicating that they boost S7P by different mechanisms.

We reasoned that decreasing the glycolytic flux by reducing the activity of phosphofructokinase (which catalyzes the rate-limiting step of glycolysis) would increase flux towards PPP and thereby boost shinorine production. However, much to our surprise, blocking G6P flux towards the PPP by deleting *ZWF1* did not hugely impact the *pfk2Δ*-mediated boost in shinorine production. After ruling out the purine salvage pathway, we hypothesized that F6P flux via the Reversed NOPPP would account for the *pfk2Δ*-mediated boost in shinorine production. Specifically, deletion of *PFK2* results in an accumulation of F6P, which then proceeds in a reverse of the canonical non-oxidative PPP reaction to produce E4P and X5P. These are in turn shunted towards S7P production through other reactions in the non-oxidative PPP (Fig. 2A). We provide multiple lines of evidence in support of the Reversed NOPPP model. Firstly, growth in the presence of a fermentable carbon source is essential for the *pfk2Δ*-mediated boost in shinorine production. Secondly, reducing F6P by overexpression of the phosphoketolase decreased the *pfk2Δ*-mediated boost. Thirdly, results from ^13^C-labelling experiments are consistent with the hypothesis that S7P is produced from F6P in the *pfk2Δ* strain. Finally, flux balance analysis experiments support the idea that the directionality of *TKL* reactions in the NOPPP is reversed in cells with reduced phosphofructokinase activity.

It has been reported that *PFK* inhibition boosts flux towards the oxidative branch of PPP in different systems. Kwak et al. [49] reported that *PFK* mutations boosted the [NADPH]/[NADP^+^] ratio even without *ZWF1* overexpression in *S. cerevisiae*, suggesting that flux towards the oxidative branch of PPP is enhanced by reducing *PFK* activity. Likewise, knockdown of *PFK* increased NADPH levels in mammalian cells [50]. Conversely, increased glycolytic flux caused by *PFK* agonists decreased NADPH levels in human immune cells [51]. Human cancer cells respond to oxidative stress by reducing glycolytic flux via degradation of *PFKB3* (phosphofructokinase/fructose bisphosphatase type-3), which boosts flux towards oxidative PPP and increases NADPH production [52]. However, the effect of blocking oxidative PPP enzymes on PPP metabolite levels in the *PFK*-inhibited cells were not tested in these studies.

Interestingly, Breitenbach-Schmitt et al. had also observed an accumulation of S7P in the *pfk2Δ* strains in yeast [53]. They too proposed a ‘reversed’ transketolase-catalyzed reaction, in which F6P is converted by transketolase and transaldolase to intermediates of the non-oxidative PPP. Such a reversal of the canonical reaction, such that the NOPPP produces ribose 5-phosphate from F6P and G3P, has been proposed in a ‘pentose-insufficiency’ model in mammals as well [48]. However, neither group provide any experimental evidence in support of their hypothesis. Moreover, the former’s original observations published about 39 years ago have not been investigated further.

Considering the importance of S7P as the common precursor for MAA production, studying its metabolism in the wild-type could prove informative. Prior work suggests that S7P is produced either through the canonical PPP, or through the dephosphorylation of SBP by Shb17 [37], with the latter pathway synthesizing about 20% of S7P in wild-type cells. The authors report an increase in flux through the Shb17 reaction in the *zwf1Δ*, *ta11Δ* and *nqm1Δ* strains, suggesting that similar carbon-labelling studies with the *PFK* mutant strains could provide additional insight on S7P metabolism. Separately, pyrophosphate-dependent *PFK* in bacteria has been reported to convert S7P to SBP ( [54, 55]); nevertheless, no evidence for a similar *PFK*-catalyzed reaction in yeast has been found.

It was interesting to note that there was a marked decrease in pentose phosphate levels in the *PFK* mutants compared to the wild-type strain (Additional File 1: Figure S11). A potential reason for this could be that the flux through the pentoses is being directed towards other growth-promoting pathways such as nucleotide and aromatic amino acid biosynthesis [56], since yeast *PFK* mutants are known to exhibit impaired growth on fermentable carbon sources [57]. This result also suggests a physiological role for the reversed NOPPP pathway, in that the reversal of the canonical direction of the first transketolase reaction (Fig. 4A) channels increased flux towards ribose production, to serve as a reserve for the production of other metabolites. A recent report from Pinson et al. [58] suggesting that an essential role of PPP in yeast is to produce phosphoribosyl pyrophosphate (PRPP) from ribose 5-phosphate further substantiates the importance of channelling flux towards R5P. Considering the implications on human health, the inducement of ribose limitation has also been proposed as a way to inhibit tumour growth [48]; the reduction in ribose levels in the *PFK* mutants of baker’s yeast suggests another potential way to achieve such a phenotype.

Reversibility of enzymatic reactions allows cells to regulate the directionality of metabolic pathways depending on the nutrient conditions. Perhaps the most well-studied example is the reversal of several of the glycolytic reactions during gluconeogenesis which occurs in the absence of glucose ( [59, 60]). Likewise, the reversibility of the transketolase and transaldolase reactions may have evolved to maintain a balance of metabolites required for energy generation (glycolysis) and DNA replication (pentose phosphate pathway). It is conceivable that there are additional cellular factors besides F6P which determine the directionality of NOPPP. A genome-wide screen to identify factors that regulate the *pfk2Δ* boost in MAA production in yeast could be informative. Screening of mutants could be facilitated by UV spectrum-based detection of MAA, which is a good proxy for S7P levels; it would also be interesting to test whether the directionality of NOPPP can be reversed in other microbial systems and mammalian cells.

Considering the significant titers required for the commercial viability of bioproducts, there is much room for further modifications to increase the amounts of MAA made in heterologous organisms such as *S. cerevisiae*. For instance, a more extensive library of promoters could be explored for the expression of the shinorine biosynthetic genes to find the most productive combinations for production. Alternatively, an extended study into the use of alternative carbon sources such as xylose [16], or a combination of carbon sources [49] could be explored. Overexpressing the transketolases *TKL1* and *TKL2* did not significantly affect the levels of shinorine production (Additional File 1: Figure S12). An alternative approach would be to engineer the specificity of transketolases to preferentially recognize F6P and G3P as substrates, to maximize the utilization of accumulated F6P in *pfk2Δ* cells and increase S7P and MAA production. The overexpression or repression of key glycolytic and PPP genes could also be an alternative approach to increase MAA production. The use of alternative heterologous hosts could also increase MAA production, for instance *Kluyveromyces marxianus*, which has a high flux through the pentose phosphate pathway [61], or the red yeast *Phaffia rhodozyma*, which contains the first two genes of the MAA biosynthetic pathway (DDGS and OMT) in its genome and produces mycosporines [62].

## Conclusion

In summary, we report a new strategy to increase the heterologous production of shinorine in *S. cerevisiae* and illuminate its mechanism of action. Using multiple lines of evidence, we demonstrate that redirection of metabolic flux from F6P towards pentose phosphates in the *pfk2Δ* mutant boosts S7P and shinorine production in *S. cerevisiae*. Despite minimal metabolic engineering steps [16], we have generated a yeast strain that produces 9-fold more shinorine than the wild-type. With further genetic and environmental perturbations, we can further increase shinorine yields and attract commercial interest. We also envision that the reversion of flux between glycolysis and NOPPP could be used to boost the production of other valuable secondary metabolites derived from S7P, such as septicidins and hygromycin B. This strategy could also work in other microbes and higher eukaryotic cells, considering the evolutionary conservation of the glycolytic and pentose phosphate pathways.

### Electronic supplementary material

Below is the link to the electronic supplementary material.

Supplementary Material 1

Supplementary Material 2

## Data Availability

No datasets were generated or analyzed during the current study.
