# Supplementary material for: Reversing the directionality of reactions between non-oxidative pentose phosphate pathway and glycolytic pathway boosts mycosporine-like amino acid production in Saccharomyces cerevisiae
Source: Microb Cell Fact. 2024 May 9;23:121. doi: 10.1186/s12934-024-02365-6 (PMC11080194; doi:10.1186/s12934-024-02365-6)
Supplement: Supplementary file 1 — Supplementary Material 1 [file 12934_2024_2365_MOESM1_ESM.docx]

## Additional File 1. Supplementary material for the article.

**Table S1.** *S. cerevisiae* strains used in this study. All strains are derived from W303 strain 1002 (*MAT*α, *ade2-1, trp1-1, can1-100, leu2-3,112, his3-11,15, ura3*) unless stated otherwise.

| **Strain** | **Genotype** | **Usage** |
| --- | --- | --- |
| YMAA1 | Delta-integration of P*_TEF1_*-NpR5600-T*_CYC1_*-P*_TEF1_*-NpR5599- T*_CYC1_*-HYG and P*_TEF1_*-NpR5598- T*_CYC1_*- P*_TEF1_*-NpR5597- T*_CYC1_*-NAT into W303 (MATα) | Figure 1B |
| YMAA2 | YMAA1 *tal1::HIS3MX6* | Figure 1G |
| YMAA3 | YMAA1 *pfk2::KANMX6* | Figure 2B |
| YMAA4 | YMAA1 *tkl2::KANMX6* | Figure S4 |
| YMAA5 | YMAA1 *tkl1::KANMX6* | Figure S4 |
| YMAA6 | YMAA1 *pfk2::KANMX6* *zwf1::HIS3MX6* | Figure 3B |
| YMAA7 | YMAA1 *zwf1::HIS3MX6* | Figure 1C |
| YMAA8 | YMAA1 *pfk2::KANMX6 tal1::HIS3MX6* | Figure 2E |
| YMAA9 | YMAA1 *tkl1::KANMX6 tkl2::HIS3MX6* | Figure 1E |
| YMAA10 | YMAA1 *pho13::HIS3MX6* | Figure S6 |
| YMAA11 | YMAA1 *nqm1::HIS3MX6* | Figure S5 |
| YMAA12 | YMAA1 *pfk1::HIS3MX6* | Figure 2C |
| YMAA13 | YMAA1 *shb17::KANMX6* | Figure S6 |
| YMAA15 | YMAA1 *pfk2::KANMX6 nqm1::HIS3MX6* | Figure S5 |
| YMAA23 | YMAA3 harbouring wild type *PFK2* in pFL38 (expression vector containing *URA3* marker)PFK2 | Figure S8 |
| YMAA24 | YMAA3 harbouring PFK2 D309T in pFL38 (expression vector containing *URA3* marker) | Figure S8 |
| YMAA25 | YMAA3 harbouring PFK2 D356S in pFL38 (expression vector containing *URA3* marker) | Figure S8 |
| YMAA26 | YMAA3 harbouring PFK2 R447S in pFL38 (expression vector containing *URA3* marker) | Figure S8 |
| YMAA27 | YMAA3 harbouring PFK2 H488S in pFL38 (expression vector containing *URA3* marker) | Figure S8 |
| YMAA28 | YMAA3 harbouring EcPFKA in pRS316 (2-micron vector containing *URA3* marker)EcPFKa | Figure S8 |
| YMAA29 | YMAA3 harbouring empty pRS316 (2-micron vector containing *URA3* marker)vector | Figure S8 |
| YMAA30 | YMAA1 *pfk2::KANMX6 prm15::HIS3MX6* | Figure S9 |
| YMAA31 | YMAA1 *pfk2::KANMX6 urh1::HIS3MX6* | Figure S9 |
| YMAA32 | YMAA1 *pnp1::HIS3MX6* | Figure S9 |
| YMAA33 | YMAA1 *prm15::HIS3MX6* | Figure S9 |
| YMAA34 | YMAA1 *atg1::HIS3MX6* | Figure S9 |
| YMAA35 | YMAA1 *urh1::HIS3MX6* | Figure S9 |
| YMAA36 | YMAA1 *pfk2::KANMX6 atg1::HIS3MX6* | Figure S9 |
| YMAA37 | YMAA1 *pfk2::KANMX6*, *tkl1::HIS3MX6, tkl2::HIS3MX6* (generated by crossing) | Figure 3D |
| YMAA38 | YMAA1 P_TEF1_-TKL1 | Figure S12 |
| YMAA39 | YMAA1 P_TEF1_-TKL2 | Figure S12 |
| YMAA40 | YMAA1 *pfk2::KANMX6 pnp1::HIS3MX6* | Figure S9 |
| YMAA41 | YMAA1 *pfk2::KANMX6 atg7::HIS3MX6* | Figure S9 |
| YMAA42 | YMAA1 *aro1::HIS3MX6* | Figure 1C |
| YMAA43 | YMAA1 *tal1::HIS3MX6*, *nqm1::HIS3MX6* | Figure S5 |
| YMAA45 | MAT-a YMAA1 *pfk2::KANMX6* *tal1:HIS3MX6, nqm1::HIS3MX6* | Figure S5 |
| YMAA49 | *tal1::HIS3MX6* | Figure 2C |
| YMAA50 | *pfk1::HIS3MX6* | Figure 2C |
| YMAA51 | YMAA1 *pfk2::KANMX6* *pfk1::HIS3MX6* | Figure 4C |
| YMAA54 | *zwf1::HIS3MX6* | Figure 1D |
| YMAA55 | *tkl1::HIS3MX6 tkl2::KANMX6* | Figure 1D |

**Figure S1.** The approximate shinorine yields (in mg/L) of the wild-type, *tal1Δ*, *pfk2Δ* and *tal1Δpfk2Δ* strains from 10 ml cultures grown overnight. Error bars represent the standard deviation (SD) of three independent experiments. Significance was determined using an unpaired *t*-test. * *p* < 0.05, ** *p* < 0.01.

**Figure S2.** (Top panel) The UV absorption profile of the shinorine-producing strain’s extract at 334 nm. (Bottom panel) The mass spectrum of the extracted ion chromatogram (EIC) peak detected at RT 6.83 min.

**Figure S3.** The UV absorption profiles of the ethanolic extracts prepared from the negative control, wild-type, *zwf1Δ*, and *zwf1Δaro1Δ* strains. The negative control strain refers to a strain which does not produce shinorine, as it has been engineered to only contain the first two genes (DDGS and OMT) of the shinorine biosynthetic pathway.

**Figure S4.** Comparison of shinorine levels in the wild-type, *tkl1∆* and *tkl2∆* strains. Error bars represent the standard deviation (SD) of three independent experiments. Significance was determined using an unpaired *t*-test. *** *p* < 0.001, **** *p* < 0.0001.

**Figure S5.** Comparison of shinorine levels in the wild-type, *pfk2∆, nqm1∆, pfk2∆nqm1∆, nqm1∆tal1∆* and *nqm1∆ tal1∆ pfk2∆* strains. Error bars represent the standard deviation (SD) of three independent experiments.

**Figure S6.** Comparison of shinorine levels in the wild-type, *shb17∆* and *pho13∆* strains. Error bars represent the standard deviation (SD) of three independent experiments.

**Figure S7.** Comparison of OD_600_ values of *S. cerevisiae* strains from an overnight culture. Error bars represent the standard deviation (SD) of three independent experiments. Significance was determined using an unpaired *t*-test. *** *p* < 0.001.

**Figure S8.** Comparison of shinorine levels produced in the *pfk2Δ* strains harbouring vectors with *PFK* variants. Error bars represent the standard deviation (SD) of three independent experiments.

**Table S2.** A description of the effects of *PFK2* mutations tested.

| **Mutation** | **Effect** |
| --- | --- |
| D309T | ATP binding |
| D356S | Substrate binding |
| R447S | Effector PEP/ADP binding |
| H488S | Substrate binding |

**Figure S9. The nucleotide degradation and ribose salvage pathway in yeast is not responsible for the *pfk2Δ* boost in shinorine production.** A) The nucleotide degradation and ribose salvage pathway in yeast. The genes *ATG7, PNP1, PRM15* and *URH1* are known to be involved in this pathway. B) Comparison of shinorine levels in the *atg1∆, pnp1∆, urh1∆* and *prm15∆* strains in the wild-type and *pfk2Δ* backgrounds. Error bars represent the standard deviation (SD) of three independent experiments.

**Figure S10. The fragmentation patterns of glycolytic/PPP metabolites.** A) G6P and F6P fragmentation patterns. Error bars represent the standard deviation (SD) of three independent experiments. Significance was determined using an unpaired *t*-test. ** *p* < 0.01, *** *p* < 0.001. B) S7P fragmentation patterns.

**Figure S11.** Comparison of the proportion of pentoses in the wild-type, *pfk1∆* and *pfk2∆* cells. Error bars represent the standard deviation (SD) of three independent experiments.

**Figure S12.** Changing the promoters of *TKL1* and *TKL2* to a strong constitutive promoter P_TEF1_ has limited effect on increasing MAA production. Error bars represent the standard deviation (SD) of three independent experiments.
